# 3D printing in anatomical lung segmentectomies: A randomized pilot trial

**DOI:** 10.1016/j.heliyon.2024.e31842

**Published:** 2024-05-26

**Authors:** Madalina Grigoroiu, Jean-François Paul, Emmanuel Brian, Philippe Aegerter, Guillaume Boddaert, Alessio Mariolo, Pierre Jorrot, Mouloud Bellahoues, Agathe Seguin-Givelet, Vittorio Perduca

**Affiliations:** a Institut Mutualiste Montsouris, Institut Du Thorax Curie-Montsouris, 42, Boulevard Jourdan, 75014, Paris, France; b Institut Mutualiste Montsouris, Département de Radiologie, 42, Boulevard Jourdan, 75014, Paris, France; c GIRCI-IDF, Cellule Méthodologie, 4, Av Richerand, 75010, Paris, France; d Université Paris-Saclay, UVSQ, Inserm, CESP U1018, 12, Av Paul-Couturier 94807, Villejuif, France; e Institut Mutualiste Montsouris, Département de Rythmologie, 42, Boulevard Jourdan, 75014. Paris, France; f Institut Mutualiste Montsouris, Département de Recherche Clinique, 42, Boulevard Jourdan, 75014, Paris, France; g Université Paris Cité, CNRS, MAP5, 44, Rue des Saint Pères, 75006, Paris, France; h Université Paris Saclay, UVSQ, INSERM, CESP U1018, « Exposome, Heredity, Cancer and Health » Team, Gustave Roussy, 12, Av Paul-Couturier, 94807, Villejuif, France

## Abstract

**Objective:**

This pilot study evaluated the impact of using a 3D printed model of the patient's bronchovascular lung anatomy on the mental workload and fatigue of surgeons during full thoracoscopic segmentectomy.

**Design:**

We performed a feasibility pilot study of a prospective randomized controlled trial with 2 parallel arms. All included patients underwent digital 3D visual reconstruction of their bronchovascular anatomy and were randomized into the following two groups: Digital arm (only a virtual 3D model was available) and Digital + Object arm (both virtual and printed 3D models were available). The primary end-point was the surgeons’ mental workload measured using the National Aeronautics and Space Administration-Task Load Index (NASA-TLX) score.

**Setting:**

Between October 28, 2020 and October 05, 2021, we successively investigated all anatomic segmentectomies performed via thoracoscopy in the Thoracic Department of the Montsouris Mutualiste Institute, except for S6 segmentectomies and S4+5 left bi-segmentectomies.

**Participants:**

We assessed 102 patients for anatomical segmentectomy. Among the, 40 were randomly assigned, and 34 were deemed analysable, with 17 patients included in each arm.

**Results:**

Comparison of the two groups, each comprising 17 patients, revealed no statistically significant difference in primary or secondary end-points. The consultation of the visual digital model was significantly less frequent when a 3D printed model was available (6 versus 54 consultations, p = 0.001). Notably, both arms exhibited high NASA-TLX scores, particularly in terms of mental demand, temporal demand, and effort scores.

**Conclusion:**

In our pilot study, 3D printed models and digital 3D reconstructions for pre-operative planning had an equivalent effect on thoracoscopic anatomic segmentectomy for experienced surgeons. The originality of this study lies in its focus on the impact of 3D printing of bronchovascular anatomy on surgeons, rather than solely on the surgical procedure.

## Graphical abstract

**Figure:**
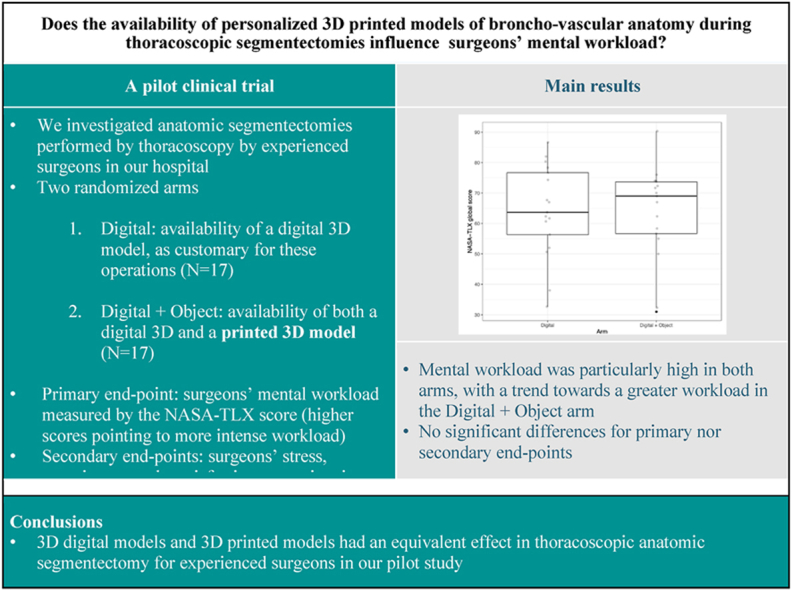

## 1 Introduction

3D printing is an emerging technology being increasingly used in clinical practice, facilitating the creation of physical models that replicate patients' anatomy for pre-operative procedure planning and intraoperative guidance in various surgical scenarios [1,2].

Sublobar anatomical resection is more technically demanding than lobectomies mainly because of a more complex bronchovascular anatomy, characterized by high variability of the vascular and bronchial anatomy, which are also very common [3]. When performing an anatomic segmentectomy, having a precise knowledge of the bronchovascular sublobar anatomy of the patient is crucial. The recognition of anatomical variants is essential in pre-operative planning to minimize pre-operative complications and optimize the success of the segmentectomy. For pre-operative planification of an anatomic resection, surgeons usually rely on their own experience in contrast-enhanced chest computed tomography (CT) 2D image interpretation and spatial reconstruction capacity, after an axial, coronary, and sagittal analysis of the images. Even experienced surgeons may encounter difficulties inidentifying some vascular variations during the surgical procedure, potentially leading to technical errors. Some retrospective studies [4,5] have suggested that, before performing minimally invasive lung anatomical segmentectomy, employing 3D CT virtual reconstruction software to perform 3-D reconstruction of anatomical structures seems to increase surgeons' confidence and contribute considerably to a safer operation. A prospective randomized study on the efficacy of three-dimensional reconstructions of bronchovascular structures on pre-operative chest CT scans in patients who are candidates for pulmonary segmentectomy surgery (PATCHES) is ongoing [6].

However, virtual 3D reconstruction is limited in practice by 2D displays. The lack of space and depth feeling may be perceived as insufficient, and an adequate 3D reconstruction of the patient's anatomy relies solely on human effort. Moreover, utilizing these models intraoperatively can lead to a risk of sepsis, since the displays are usually not sterile.

Having a personalized 3D printed model of the patient's lung bronchovascular anatomy available in the operating field allows the surgeon to refer to it at any time, aiding in recalling or understanding the anatomy during surgery without compromising sterility. This capability could decrease mental effort and the time required by surgeons to understand the anatomy before and during the procedure, thereby increasing their confidence and the safety of the surgical procedure. Consequently, surgeons may experience reduced fatigue at the end of the procedure and be better prepared for the subsequent operation.

This pilot randomized controlled trial aimed to evaluate the influence of incorporating personalized 3D printed models of pulmonary bronchovascular anatomy of a patient on mental workload and fatigue of surgeons during a full thoracoscopic anatomical segmentectomy.

## 2 Materials and methods

### 2.1 Study design

Between October 28, 2020 and November 05, 2021, we successively enrolled all patients undergoing anatomic segmentectomies performed by full thoracoscopy in our department, excluding S6 segmentectomies, S4+5 left bi-segmentectomies and cases with insufficient time for obtaining 3D printed models, which were outsourced to an external company. We performed a prospective randomized controlled pilot trial with two parallel arms.

- Digital arm
- Digital + Object arm.

**Fig. 1:**
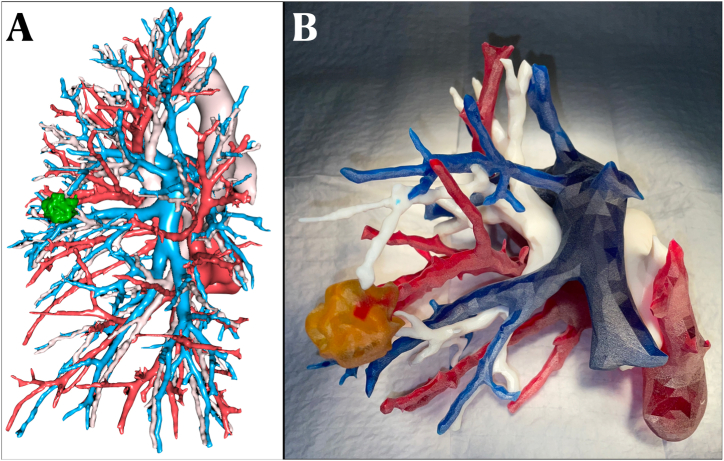
Preoperative bronchovascular anatomy 3D reconstruction. (A) virtual 3D reconstruction model: bronchial tree in yellow, pulmonary arteries in blue, pulmonary veins in red, tumour in green and safety margins in light green. (B) print 3D reconstruction model: bronchial tree in white, pulmonary arteries in blue, pulmonary veins in red, and tumour in yellow. (For interpretation of the references to colour in this figure legend, the reader is referred to the Web version of this article.)

**Fig. 2:**
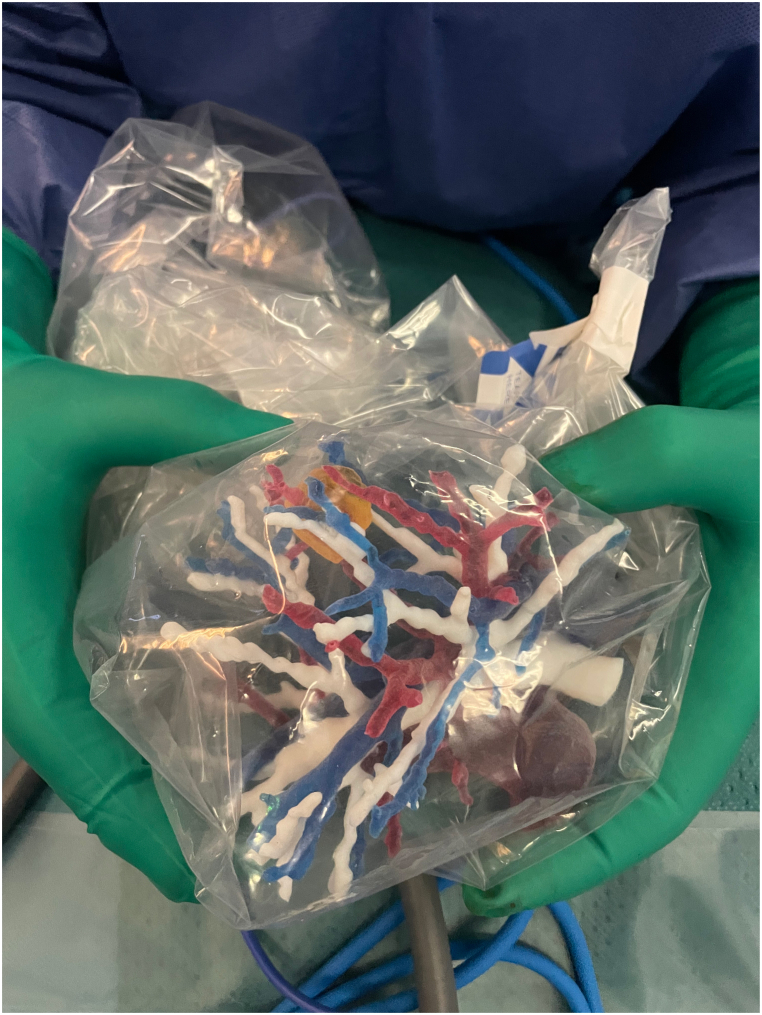
3D printed bronchovascular anatomy in a sterile bag on the operating field.

In both arms, a 3D digital model (Fig. 1A) of the patient's pulmonary anatomy, created using the Visible Patient Planning (VP) software (VISIBLE PATIENT™ Planning Solution, Strasbourg, France), was made available to the surgeon several days before surgery and was accessible on a screen in the operating room, consistent with our department's standard practice for this type of lung resection [7]. In the Digital + Object arm, surgeons were additionally provided with a 1:1 scale 3D printed models (Fig. 1B) depicting the patient's lung bronchovascular anatomy. These physical models were placed in a transparent sterile bag on the operating field (Fig. 2) and provided to the surgical team no later than the day before surgery. In this arm, the visual 3D digital model was available as in the Digital arm, but its use during surgery was permitted only after consulting the 3D printed model, considered a ‘failure’ of the 3D printed model.

The procedures were all performed by five senior surgeons, all experienced in this type of surgery. With more than 850 procedures to date [8,9], our department specializes in full thoracoscopic anatomical segmentectomies, which represent 25–30 % of our major lung resections.

### 2.2 Endpoints

The primary end-point was surgeon's mental load as measured by the National Aeronautics and Space Administration-Task Load Index (NASA-TLX) score, a widely used measure of mental workload [10,11], including in medical contexts [12]. It is a multidimensional rating scale with the following six bipolar dimensions: mental demand (MD); physical demand (PD); time demand (TD); self-performance (P); effort (E); and frustration (F). The dimensions thus reflect tasks (DM, PD, and TD), performance (P), and behavioural factors (E and F). The NASA-TLX score is calculated through a self-questionnaire, yielding a global load score between 0 and 100 and by sub-dimensions, with higher scores indicating higher mental loads of subjective perceptions. The NASA-TLX mental load self-questionnaire was administered to the surgeon immediately at the end of each intervention.

Secondary end-points included surgeon's stress during surgery, work attention, and overall satisfaction at the end of surgery.

To assess acute stress, the surgeons wore a Holter device throughout the surgical procedure. The recordings were analysed by a rhythmologist according to several parameters: minimum, average, and maximum heart rates, percentage of extrasystoles, and the duration of time with a heart rate >100 beats/minute. None of the surgeons had any cardiac condition. The normal physiologic response to stress results in increased cardiac activity (heart rate activity and arrhythmia) [13,14].

For the evaluation of the surgeon's attention at the end of the surgery, we used the following two simple tests, commonly used in medical-surgical contexts [15,16].

- The d2 test of attention is a validated test for evaluating selective attention (ability to select relevant information in the presence of distractors) and concentration [17]. A commercially available computer-assisted form (Hogrefe, Göttingen, Germany) for the d2-R test was chosen to eliminate testing bias and time overrun. Scoring of the d2 test included concentration performance (CC), representing the capacity of concentration; the total number of target items processed, including the omitted ones (CCT); and percentage of errors (E %), defined as the total number of errors × 100/CCT that reflects performance accuracy. The scores were standardized to standard scores (NS), ranging from 20 to 80, with an average score of 50.
- The EncephalApp Stroop test was used to evaluate psychomotor speed and cognitive flexibility [18]. Norms for EncephalApp Stroop scores in healthy populations were defined in the United States [19,20]. We used a free digital app for smartphones, which was easy to administer and simple to score and interpret [20]. The specific outcomes of the EncephalApp Stroop test included the total time for five correct runs in the ‘off’ (OffTime) and ‘on’ (OnTime) states; the number of runs needed to complete the five correct ‘off’ runs and five correct ‘on’ runs. The test of cognitive processes controlling for psychomotor speed was obtained by adding the OffTime to the OnTime, which has been found in prior Encephal App Stroop test studies to be identified as the best discriminator between participants [21]. OnTime minus OffTime was assessed to evaluate cognitive flexibility independent of psychomotor speed [22]. None of the participating surgeons had red-green colour blindness.

To assess surgeon subjective satisfaction at the end of the surgery, we used a 5-point Likert scale (1 = totally dissatisfied; 2 = dissatisfied; 3 = neither dissatisfied nor satisfied; 4 = satisfied; and 5 = extremely satisfied).

Other secondary end-points included the duration of the anatomical segmentectomy (operation time) calculated starting from skin incision to the completion of segmentectomy, the number of sterility errors due to glove changes when consulting the virtual 3D models, the number of consultations of the VP model, the number of consultations of the 3D printed model, and the number of consultations of the VP model when a 3D printed model was available. These variables were recorded by an external observer present in the operating room throughout the surgical procedure.

Subgroup analyses were pre-specified to compare all four possible combinations between the intervention (Digital, Digital + Object) and segmentectomy complexity (complex, simple). We defined segmentectomies with at least one dihedral angle between the inter-segmental planes as ‘complex’.

For this pilot study, we had no previous measurements in this context. We considered that a standardized effect size of 1 on mental load score would be the target; therefore, a two-sided risk of error of 5 % and a power of 80 % indicated the inclusion of 17 patients in each arm, i.e., 40 patients in total, taking into account 15 % of non-evaluable patients.

For comparison, the randomized trial of Chowriappa [23] showed a standardized effect size of 0.7 (or even 1 in intra-subject comparison) on mental load (measured using the NASA-TLX) when using an augmented reality system.

Randomization was conducted centrally using a web server.

### 2.3 Statistical analysis

Quantitative outcomes are described as median and the first and third quartiles (Q1 and Q3 respectively). The distributions of continuous outcomes are represented with boxplots.

Analysis was performed on per-protocol populations. Comparisons between the two arms were performed using two-sample Wilcoxon rank sum tests for numeric outcomes and Fisher's exact tests for categorical outcomes.

For subgroup analyses, comparisons based on all possible combinations between arms and type of segment complexity were performed using Kruskal–Wallis tests. For significant Kruskal–Wallis tests, post-hoc analyses based on pairwise Wilcoxon tests were performed to determine the statistical difference between the groups; p-values of such tests were adjusted according to the Bonferroni correction. All tests were two-tailed and all statistical analyses were conducted using R, version 4.1.2 [24] and in particular with packages in the tidyverse collection [25] and gtsummary [26].

### 2.4 Ethics

This project is an evaluation in the field of health CEPAR 2019-04 and is registered on ClinicalTrials.gov: Protocol Record THOR-02-2019, Identifier: NCT05695404.

In the Digital and Digital + Object arms, surgical procedures were conducted as per standard practice. In the Digital + Object arm, the surgeons were free to consult virtual anatomical models, as they usually did if they considered that consulting only the 3D printed model was insufficient.

## 3 Results

**Fig. 3:**
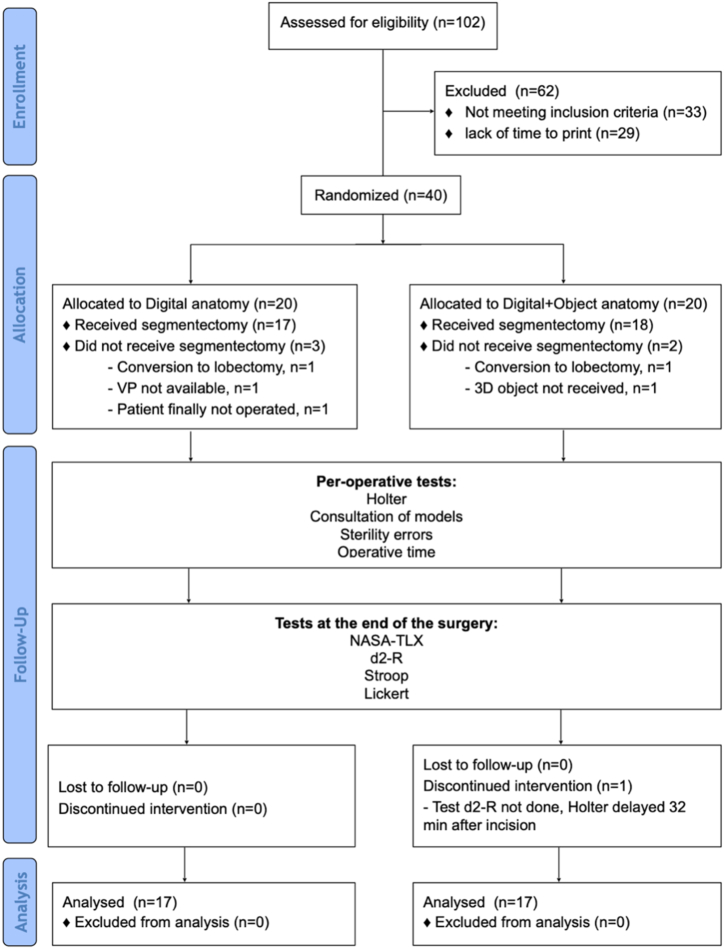
Study flow chart. VP = Visible Patient Planning software; NASA-TLX = National Aeronautics and Space Administration-Task Load Index.

Between October 28, 2020 and November 05, 2021 we assessed 102 patients for anatomical segmentectomy. A total of 40 patients were randomly allocated, of which 34 were deemed analysable, with 17 patients included in each arm (Fig. 3).

The baseline characteristics of the patients were well balanced between the two groups except for the age of the patients (Table 1).

**Table 1:** Baseline characteristics of the patients.

Characteristic	Digital, N = 17^a^	Digital + Object, N = 17^a^	*P*^b^
**Age, years**	61 (53–66)	68 (64–74)	0.03
**Gender** Female Male	7 (41 %) 10 (59 %)	9 (53 %) 8 (47 %)	0.70
**Segment type** **Simple** S1-3 L S4-5 + S3 L S1+2 S1a+2 S1b+2 S2 **Complex** S6+9 S7+8 S6+10 S^6^+S^9a^ + S^8a^ S8 S8+9	14 (82 %) 7 0 4 0 1 2 3 (18 %) 0 0 1 0 1 1	12 (71 %) 4 1 5 2 0 0 5 (29 %) 1 2 1 1 0 0	0.70
**Surgeon** AS DG EB GB MG	5 (29 %) 3 (18 %) 2 (12 %) 2 (12 %) 5 (29 %)	5 (29 %) 3 (18 %) 2 (12 %) 1 (5.9 %) 6 (35 %)	>0.99

a Median (interquartile interval Q1-Q3); n(%).

b Wilcoxon rank sum exact test; Pearson's Chi-square test; Fisher's exact test; L = left.

All the results comparing the analysed characteristics between the two arms (Digital and Digital + Object) are summarized in Table 2.

**Table 2:** Results comparing the analysed characteristics between the patients in the two arms and results of the subgroup analysis comparing complex versus simple segmentectomies for each arm.

Characteristic	Digital N = 17^a^	Digital + Object N = 17^a^	P^2^	Complex segments		Simple segments		P^3^
				Digital N = 3^a^	Digital + Object N = 5^a^	Digital N = 14^a^	Digital + Object N = 12^a^	
**NASA-TLX**								
Mental demand	85 (80, 85)	90 (80, 90)	0.14	85 (85, 90)	90 (90, 95)	85 (80, 85)	82 (79, 90)	0.025
Physical demand	65 (35, 85)	70 (55, 85)	0.70	35 (30, 60)	70 (65, 75)	72 (50, 85)	65 (34, 86)	0.70
Temporal demand	80 (70, 85)	75 (65, 90)	>0.99	75 (75, 82)	90 (75, 90)	80 (66, 84)	70 (46, 90)	0.60
Performance	10 (10, 25)	15 (10, 20)	0.70	10 (8, 52)	15 (5, 20)	12 (10, 24)	15 (10, 26)	>0.99
Effort	75 (65, 85)	75 (65, 85)	0.70	75 (65, 82)	85 (70, 85)	80 (66, 85)	72 (65, 86)	>0.99
Frustration	35 (10, 50)	20 (10, 50)	0.40	20 (12, 35)	20 (10, 20)	40 (11, 61)	22 (9, 50)	0.70
Weighted rating (global score)	64 (56, 77)	69 (57, 74)	0.90	68 (59, 75)	70 (69, 74)	63 (57, 76)	65 (56, 73)	>0.99
**d2-R**								
Concentration performance (CC)	256 (202, 274)	266 (226, 282)	0.40	256 (228, 258)	280 (278, 283)	258 (209, 278)	244 (220, 270)	0.13
NS CC	68 (54, 74)	71 (60, 75)	0.40					
Speed performance (CCT)	265 (214, 278)	278 (260, 285)	0.40	265 (234, 268)	288 (282, 291)	264 (224, 286)	264 (240, 280)	0.10
NS CCT	65 (52, 68)	68 (64, 69)	0.30					
Percentage of errors (E%)	2.80 (2.00, 4.10)	2.70 (1.40, 6.20)	0.80	3.40 (2.70, 3.75)	2.70 (2.10, 2.70)	2.80 (2.02, 4.68)	3.65 (1.10, 8.03)	0.80
NS E%	59 (55,62)	60 (51, 65)	0.90					
**Stroop Test**								
OffTime	54.2 (50.4, 60.4)	54.9 (50.4, 59.0)	0.80	50.0 (49.7, 51.4)	54.4 (49.8, 54.5)	54.5 (52.0, 62.3)	56.2 (53.4, 60.0)	0.20
OnTime	57 (55, 61)	55 (53, 62)	0.30	56 (56, 57)	55 (53, 55)	58 (54, 63)	57 (54, 64)	0.40
No. of off runs	5.00 (5.00, 6.00)	5.00 (5.00, 5.00)	0.20	5.00 (5.00, 5.50)	5.00 (5.00, 6.00)	5.00 (5.00, 6.00)	5.00 (5.00, 5.00)	0.40
No. of on runs	5.00 (5.00, 6.00)	5.00 (5.00, 5.00)	0.11	6.00 (5.50, 6.00)	5.00 (5.00, 5.00)	5.00 (5.00, 5.00)	5.00 (5.00, 5.00)	0.11
OffTime + OnTime	110 (107, 121)	109 (105, 119)	0.70	106 (105, 108)	107 (105, 109)	112 (109, 121)	114 (107, 122)	0.30
OnTime-Off-Time	4.9 (−0.1, 5.7)	2.8 (0.4, 5.8)	0.90	5.7 (5.3, 5.8)	0.4 (0.1, 2.9)	2.2 (−0.3, 5.4)	3.1 (1.0, 7.1)	0.50
**Operative time (minutes)**	Median 124 (93, 159)	Median 126 (104, 161)	0.70	159 (146, 190)	161 (145, 220)	114 (86, 154)	113 (94, 129)	0.049
	Mean 129 (63, 221)	Mean 141 (60, 274)						
**No.** o**f consultations**^d^								
								
Digital or Object (according to arm)^g^	2.00 (0, 16)	2.00 (0, 4)	0.80	3 (0, 16)	2 (1, 3)	2 (0, 13)	2 (0, 4)	0.80
Digital and Object (according to arm)^h^	2.00 (0, 16)	2.00 (0, 6)	0.60	3 (0, 16)	2 (1, 6)	2 (0, 13)	2 (0, 5)	0.70
**No of sterility errors**	0	0						
**Holter data**								
HRmin (beats/min)	66 (58, 68)	68 (64, 74)		52 (47, 57)	73 (67, 74)	66 (60, 69)	68 (63, 69)	
Unknown	1	3	0.20	1	0	0	3	0.13
HRmoy (beats/min)	85 (80, 90)	88 (82, 93)		76 (73, 80)	94 (89, 95)	86 (81, 92)	85 (77, 90)	
Unknown	1	3	0.50	1	0	0	3	0.20
HRmax (beats/min)	112 (110, 120)	110 (105, 117)		112 (111, 112)	110 (107, 121)	114 (110, 121)	109 (105, 116)	
Unknown	1	3	0.20	1	0	0	3	0.50
Extrasystoles (%)	0.10 (0.00, 0.60)	0.00 (0.00, 0.00)		0.20 (0.10, 0.30)	0.00 (0.00, 0.00)	0.10 (0.00, 0.60)	0.00 (0.00, 0.00)	
Unknown	1	3	0.009	1	0	0	3	0.069
Time (ms) HR > 100 beats/min	2 (0, 5)	3 (0, 12)		2 (1, 3)	15 (0, 29)	2 (0, 6)	3 (0, 3)	
Unknown	1	3	>0.99	1	0	0	3	>0.99

Characteristic	DigitalN = 17^e^	Digital + ObjectN = 17^e^	p-value^f^	Complex segments		Simple segments		p-value^f^
				DigitalN = 3^e^	Digital + ObjectN = 5^e^	DigitalN = 14^e^	Digital + ObjectN = 12^e^	
**Surgeon satisfaction**			0.80					0.64
Extremely unsatisfied	0 (0 %)	0 (0 %)		0 (0 %)	0 (0 %)	0 (0 %)	0 (0 %)	
Unsatisfied	2 (12 %)	2 (12 %)		0 (0 %)	0 (0 %)	2 (14 %)	2 (17 %)	
Neutral	2 (12 %)	1 (5.9 %)		0 (0 %)	0 (0 %)	2 (14 %)	1 (8.3 %)	
Satisfied	8 (47 %)	6 (35 %)		3 (100 %)	1 (20 %)	5 (36 %)	5 (42 %)	
Extremely satisfied	5 (29 %)	8 (47 %)		0 (0 %)	4 (80 %)	5 (36 %)	4 (33 %)	

Median (interquartile interval Q1-Q3).

Wilcoxon rank sum exact test.

Kruskal-Wallis rank sum test.

Median (min-max range).

n(%).

Fisher's exact test.

Number of consultations of the 3D digital model in the Digital arm and of the 3D printed model in the Digital + Object arm.

Number of consultations of the 3D digital model in the Digital arm and of the 3D digital or printed models in the Digital + Object arm. HR = Heard rate.

### 3.1 Primary end-point

**Fig. 4:**
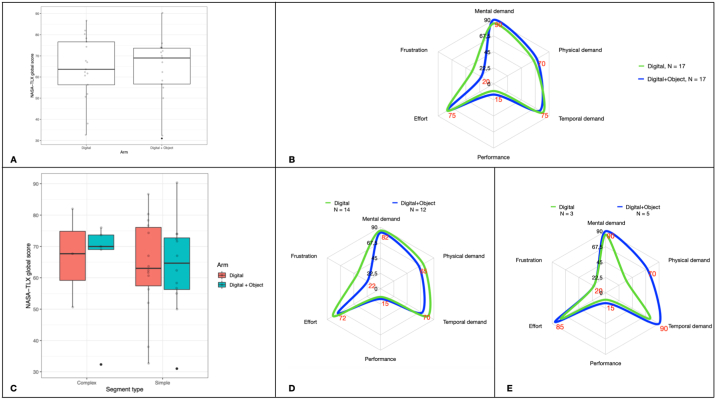
The NASA-TLX scores according to arm and segment complexity: (A) NASA-TLX global score; (B) NASA-TLX global score according to its six dimensions and arms of the study; (C) NASA-TLX global scores according to the complexity of the segmentectomies; (D) NASA-TLX scores of the simple segmentectomies; (E) NASA-TLX scores of the complex segmentectomies.

A trend towards a greater mental workload was observed in favour of the Digital + Object arm than for the Digital arm (Fig. 4A–E); however, the difference was not significant (Table 2).

### 3.2 Secondary end-points

Regarding surgeons’ stress during surgery, no significant difference was observed between the two arms for any of the parameters analysed, except for the presence of more extrasystoles in the Digital arm than in the Digital + Object arm (0.1 % vs. 0.0 %, respectively; p = 0.009). This difference was no longer significant after Bonferroni correction.

No significant difference was observed between the two arms for surgeons' attention to work (d2-R test and STROOP test) or subjective satisfaction at the end of the surgical procedure (Table 2).

The operation time was similar and no sterility errors were recorded. No intraoperative complications were recorded in either arm, including vascular events or other adverse events. No conversion to thoracotomy was required.

No difference was observed in the frequency of model consultation (Digital and 3D printed model) between the two arms. In both arms, we noted two types of consultation of the digital model by the surgeon as follows: a quick ‘take a look’ without manipulation of the screen by the surgeon and a ‘deeper exploration’ that required screen manipulation. In this situation, the surgeon changes the sterile gloves twice for each consultation. In the Digital arm, the model was consulted 54 times (average 2 times/intervention, range 0–16 times/intervention). For five patients (29 %) in this arm, consulting the digital model was not deemed necessary. In the Digital + Object arm, the 3D printed model was consulted 33 times (p = 0.6), with an average of 2 times/intervention (between 0 and 4 times/intervention). For one patient (6 %) in this arm, no model consultation was required during surgery. In contrast, for 12 patients (71 %), consultation of the 3D printed model alone was considered sufficient. Additional consultation of the Digital model was deemed necessary for four patients (23 %), with a total of six consultations. In this group, only one patient had undergone complex segmentectomy (right S6+10), and the three others underwent right S1+2 segmentectomy. For two patients, all 3D printed model consultations (3 times and 1 time, respectively) were followed by a ‘deeper exploration’ consultation of the digital model. For one patient, the 3D printed model was consulted four times with only one ‘take a look’ consultation of the VP model. For the last patient in this group, the 3D printed model was consulted twice, with only one ‘take a look’ consultation of the VP model.

In the Digital arm, 19 screen touching and 38 pairs of sterile gloves were used, compared to 8 pairs of gloves used for 4 screen manipulation in the Digital + Object arm (p = 0.02).

Finally, consultation of the digital model was significantly different in the Digital + Object arm (6 consultations) than in the Digital arm (54 consultations) (p = 0.013).

All results of the subgroup analyses comparing complex (8 patients) versus simple (26 patients) segmentectomies for each arm are summarized in Table 2. Additionally, the NASA-TLX global score is presented in Fig. 3. The mental demand component of the NASA-TLX and operation time was significantly different across the four groups before Bonferroni correction. According to post-hoc analyses, the only groups to exhibit significant difference after Bonferroni correction were the simple segments group in the Digital arm and the complex segment group in the Digital + Object arm with respect to the NASA-TLX mental demand (p = 0.016), being higher in complex segmentectomies (Table 3).

**Table 3:** Post-hoc pairwise comparisons using Wilcoxon rank sum test with continuity correction; Bonferroni adjusted p-values.

	Complex segments, Digital	Complex segments, Digital + Object	Simple segments, Digital
NASA-TLS mental demand			
**Complex, Digital** + **Object**	1.00	–	–
**Simple, Digital**	0.46	0.02	–
**Simple, Digital** + **Object**	1.00	0.39	1.00
Operation length			
**Complex, Digital** + **Object**	1.00	–	–
**Simple, Digital**	0.72	0.20	–
**Simple, Digital** + **Object**	0.61	0.16	1.00

An average of 3 days was necessary to create the digital model and.stl file needed for 3D printing. The mean duration of execution and delivery of the 3D printed models was 7 days. Because of this delay, we could not include 29 eligible patients.

## 4 Discussion

3D printing is a modern technology that has penetrated the medical field over recent years. Compared to the virtual reconstruction of an anatomical model from CT or magnetic resonance images displayed on a 2D screen, the 3D printed models can provide a realistic representation of the spatially complex bronchovascular anatomy. Surgeons can more clearly visualize and experience the three-dimensional relationships of each structure. The application of this technology in thoracic surgery is limited, with only a few studies reporting its use in thoracoscopic pulmonary segmentectomy [27,28].

We conducted a pilot prospective randomized controlled trial with two arms of patients undergoing anatomic segmentectomy performed via thoracoscopy. We hypothesized that the use of 3D-printed models would affect surgeons' mental workload and fatigue. Our study revealed no difference between the two arms on any criterion.

Other articles have reported similar results [29,30]. Notably, these studies have evaluated the use of 3D printing by evaluating patient outcomes, whereas we chose to evaluate its impact on surgeons. Liu et al. [29] did not find any difference in a retrospective analysis involving 124 patients undergoing thoracoscopic anatomical segmentectomy performed by experienced surgeons (similar to our study) when comparing intraoperative blood loss, operation time, conversion to thoracotomy, post-operative chest tube duration, post-operative complications, and post-operative hospital stay.

Mental workload can be thought of as the amount of attention an operator can direct to a task at any given moment or the difference between task demands and available attentional resources [31]. A highly mentally demanding task leaves little or no spare attentional capacity for new or unexpected events, and results in inferior task performance and a higher likelihood of errors [32,33]. In our study, we noted particularly high scores for mental demand, temporal demand, and effort. The weighted rating was also high in both arms (mean NASA-TLX scores = 64 in the Digital arm and 69 in the Digital + Object arm), reaching 77, with minimum scores remaining above 56. In other industries (eg, aviation), the NASA-TLX score has been adopted as a meaningful measure of mental workload, and global NASA-TLX load scores over 50 have been associated with reduced performance [34]. The exact workload thresholds that result in declines in surgeons' health and patients’ safety are subject to debate [35]. However, investigators tentatively observed that mental demand was a major source of workload, and NASA-TLX workload scores over 50 led to increased errors during clinical tasks [36]. The observed high NASA-TLX scores may raise concerns regarding the increased risk of errors during this type of intervention. It is possible that when faced with high workload levels, physicians may consciously or subconsciously make adjustments, such as taking more time to make decisions or consulting with other physicians or devices, to ensure optimal performance [36]. As operations become more complex and reliant on technology, the mental and physical demands on surgeons and their teams are expected to rise accordingly.

The d2-R test has been proposed as a particularly useful tool for measuring attention and concentration [37]. When attention is coupled with concentration, it is also called ‘focused attention’ [38]. A high ability to concentrate (high CC score) is generally associated with fast and accurate processing of the test. The NS CC scores exceeded 65 in both arms, without any significant difference between the two. Only 7 % of the normative sample scored over 65. This suggests that although full thoracoscopic segmentectomies are complex procedures involving a high mental demand, they do not seem to affect surgeons' attention capacity.

Interestingly, in the Digital + Object arm, for most patients (71 %), consulting only the 3D printed model was deemed sufficient. The surgeons required to consult the VP model in only four patients. The reasons for the 3D printed model ‘failure’ in these cases are difficult to elucidate. No specific issue was reported for these four operations, as evidence by comparable mean operation time and NASA-TLX scores.

In the Digital arm, the number of times the digital model was consulted through screen touching was significantly larger than that in the Digital + Object arm. This manoeuvre poses a risk because screens are not sterile. Although in our study, the external observer collecting the data did not notice any sterility error, this practice warrants caution.

Other studies have focused on the contribution of surgical anatomy printing technologies on so-called complex anatomical segmentectomies. Qiu et al. [30] retrospectively reviewed data of 298 cases who underwent anatomical segmentectomies. In the complex segmentectomy group, they observed significantly shorter operation time and lesser intraoperative bleeding with a 3D printed model present in the operating room (but not in the operating field) in comparison with ‘3D-reconstruction’-Digital group. In this study, no significant difference was observed between the two arms in terms of simple segmentectomies. Although there is currently no academic definition of complex segmentectomies, most authors agree that complex anatomical segmentectomies involve the resection of two or more inter-segmental planes. In our study, 26 simple segmentectomies and 8 complex segmentectomies were equally balanced between the two arms (Table 1). The subgroup analyses, which compared the endpoints for all possible combinations of the segmentectomy complexity and intervention, revealed that surgeons' mental demand, assessed using the NASA-TLX, was higher for simple segmentectomy in the complex Digital + Object arm than in the Digital arm (90 versus 85, respectively; p = 0.016). However, it remains uncertain whether the 3D printed model plays any role in reducing this mental demand or not.

The limitations of our study are mainly related to the small number of patients included and the mono-centric design with experienced surgeons in full thoracoscopic anatomical segmentectomies. Indeed, this is a pilot feasibility study. A new prospective randomized study with a sufficient number of patients would be necessary to validate these initial findings and to determine whether 3D printing technology offers benefits, including those for younger surgeons.

With regards to 3D printing technology, we believe that the logistics of setting up a clinical 3D printing service, including the costs of purchasing and maintaining a 3D printer, are the main limitations to its use. Moreover, despite the outsourcing to highly specialized external companies for both radiological image segmentation and 3D printing, each case requires an average of 10 days before the 3D anatomic model is available, even though it is now feasible to obtain a 3D printed model without going through 3D virtual segmentation, which shortens the time. The use of automatic software for the segmentation of the bronchovascular tree and the possession of its own 3D printer, could drastically reduce the manufacturing time to 24 h. Another limitation is that the sterilization procedures for this type of complex resin or silicon structures have not yet been described. In our study, we used sterile, transparent plastic bags to place printed models on the operating field. The visibility was acceptable but not optimal, in the semi-dark operating rooms typical of thoracoscopic surgery, which may explain the need for consulting the virtual model in addition to the printed model for four patients. Nevertheless, consulting the 3D printed model seems to be sufficient since the VP model was significantly less frequently consulted in the Digital + Object arm than in the Digital arm, even though we did not measure the duration of consultation of the different models available across the arms and procedural complexities, which is another limitation.

## 5 Conclusion

Our pilot study suggests that 3D printed models could be valuable for preoperative planning and intraoperative guidance in full thoracoscopic anatomic segmentectomy for experienced surgeons. Their availability during surgery offers some practical advantages, such as avoiding the need to change gloves and handling non-sterile screens. Potential applications are currently limited by the cost, manufacturing time, and the need to put the 3D printed models in a sterile bag in the operative field. Considering these limitations, it does not seem appropriate to create 3D printed models for all sublobar anatomical lung resections at present. However, using this technology on an ad hoc basis, depending on the complexity of the lesions and/or that of the surgical procedure, will likely increase our current knowledge and improve the ability of thoracic surgeons to plan the appropriate surgical strategy. This strategic approach could serve as a catalyst for advancing the development of 3D print models in thoracic surgery in the future.

## Ethics statement

The authors declare that the work described has been carried out in accordance with the Declaration of Helsinki of the World Medical Association revised in 2013 for experiments involving humans as well as in accordance with the EU Directive 2010/63/EU for animal experiments.

The authors declare that they obtained a written informed consent from the patients and/or volunteers included in the article and that this report does not contain any personal information that could lead to their identification.

## Funding

This work was supported by the Fondation de l’Avenir, Paris, France [AP-RM-19-012, 2019].

## Disclosures of all co-authors

The authors report no proprietary or commercial interest in any product mentioned or concept discussed in this article.

## Data availability statement

The data associated with this study have not been deposited in a publicly accessible repository for reasons of confidentiality. However, the data will be made available upon reasonable request to the corresponding author.

## CRediT authorship contribution statement

**Madalina Grigoroiu:** Writing – review & editing, Writing – original draft, Validation, Supervision, Project administration, Funding acquisition, Formal analysis, Data curation, Conceptualization. **Jean-François Paul:** Writing – review & editing, Validation, Funding acquisition, Conceptualization. **Emmanuel Brian:** Validation, Investigation. **Philippe Aegerter:** Writing – review & editing, Visualization, Validation, Methodology, Conceptualization. **Guillaume Boddaert:** Validation, Investigation. **Alessio Mariolo:** Validation. **Pierre Jorrot:** Validation, Investigation. **Mouloud Bellahoues:** Validation, Investigation, Data curation, Conceptualization. **Agathe Seguin-Givelet:** Validation, Investigation. **Vittorio Perduca:** Writing – review & editing, Writing – original draft, Visualization, Validation, Formal analysis.

## Declaration of competing interest

The authors declare the following financial interests/personal relationships which may be considered as potential competing interestsMadalina Grigoroiu reports financial support was provided by Fondation de l’Avenir pour la Recherche Médicale Appliquée. If there are other authors, they declare that they have no known competing financial interests or personal relationships that could have appeared to influence the work reported in this paper.

## References

[1] Mitsouras D., Liacouras P., Imanzadeh A., Giannopoulos A.A., Cai T., Kumamaru K.K., George E., Wake N., Caterson E.J., Pomahac B., Ho V.B., Grant G.T., Rybicki F.J. (2015). Medical 3D printing for the radiologist. Radiographics.

[2] Matsumoto J.S., Morris J.M., Foley T.A., Williamson E.E., Leng S., McGee K.P., Kuhlmann J.L., Nesberg L.E., Vrtiska T.J. (2015). Three-dimensional physical modeling: applications and experience at mayo clinic. Radiographics.

[3] Shimizu K., Nagashima T., Ohtaki Y., Obayashi K., Nakazawa S., Kamiyoshihara M. (2016). Analysis of the variation pattern in right upper pulmonary veins and establishment of simplified vein models for anatomical segmentectomy. Gen Thorac Cardiovasc Surg.

[4] She X.W., Gu Y.B., Xu C., Li C., Ding C., Chen J. (2018). Three-dimensional (3D)- computed tomography bronchography and angiography combined with 3D video-assisted thoracic surgery (VATS) versus conventional 2DVATS anatomic pulmonary segmentectomy for the treatment of non-small cell lung cancer. Thorac Cancer.

[5] Wu Z., Huang Z., Qin Y., Jiao W. (2022). Progress in three-dimensional computed tomography reconstruction in anatomic pulmonary segmentectomy. Thorac Cancer.

[6] Zaraca F., Kirschbaum A., Pipitone M.D., Bertolaccini L., PATCHES study group (2023 Sep 16). Prospective randomized study on the efficacy of three-dimensional reconstructions of bronchovascular structures on preoperative chest CT scan in patients who are candidates for pulmonary segmentectomy surgery: the PATCHES (Prospective rAndomized sTudy efficaCy of tHree-dimensional rEconstructions Segmentecomy) study protocol. Trials.

[7] Gossot D., Lutz J., Grigoroiu M., Brian E., Seguin-Givelet A. (2016). Thoracoscopic anatomic segmentectomies for lung cancer: technical aspects. J. Vis. Surg..

[8] Lutz J.A., Seguin-Givelet A., Grigoroiu M., Brian E., Girard P., Gossot D. (2019). Oncological results of full thoracoscopic major pulmonary resections for clinical Stage I non-small-cell lung cancer. Eur. J. Cardio. Thorac. Surg..

[9] Seguin-Givelet A., Lutz J., Brian E., Grigoroiu M., Gossot D. (2018). Traitement chirurgical des cancers bronchiques non à petites cellules (CBNPC) de stade précoce par segmentectomie à thorax fermé : résultats préliminaires [Surgical treatment of early stage non-small cell lung cancer by thoracoscopic segmental resection]. Rev. Mal. Respir..

[10] Hart S.G., Staveland L.E., Hancock P.A., Meshkati N. (1988). Human Mental Workload.

[11] Wilson M.R., Poolton J.M., Malhotra N., Ngo K., Bright E., Masters R.S. (2011). Development and validation of a surgical workload measure: the surgery task load index (SURG-TLX). World J. Surg..

[12] Dias R.D., Ngo-Howard M.C., Boskovski M.T., Zenati M.A., Yule S.J. (2018). Systematic review of measurement tools to assess surgeons' intraoperative cognitive workload. Br. J. Surg..

[13] Campbell DA Jr, Sonnad S.S., Eckhauser F.E., Campbell K.K., Greenfield L.J. (2001). Burnout among American surgeons. Surgery.

[14] Arora S., Sevdalis N., Nestel D., Woloshynowych M., Darzi A., Kneebone R. (2010). The impact of stress on surgical performance: a systematic review of the literature. Surgery.

[15] Weiss P., Kryger M., Knauert M. (2016). Impact of extended duty hours on medical trainees. Sleep Health.

[16] Amirian I., Andersen L.T., Rosenberg J., Gögenur I. (2014). Laparoscopic skills and cognitive function are not affected in surgeons during a night shift. J. Surg. Educ..

[17] Brickenkamp R., Zillmer E. (1998).

[18] Bajaj J.S., Hafeezullah M., Franco J., Varma R.R., Hoffmann R.G., Knox J.F., Hischke D., Hammeke T.A., Pinkerton S.D., Saeian K. (2008). Inhibitory control test for the diagnosis of minimal hepatic encephalopathy. Gastroenterology.

[19] Scarpina F., Tagini S. (2017). The stroop color and word test. Front. Psychol..

[20] Allampati S., Duarte-Rojo A., Thacker L.R., Patidar K.R., White M.B., Klair J.S., John B., Heuman D.M., Wade J.B., Flud C., O'Shea R., Gavis E.A., Unser A.B., Bajaj J.S. (2016). Diagnosis of minimal hepatic encephalopathy using stroop EncephalApp: a multicenter US-based, norm-based study. Am. J. Gastroenterol..

[21] Bajaj J.S., Heuman D.M., Sterling R.K., Sanyal A.J., Siddiqui M., Matherly S., Luketic V., Stravitz R.T., Fuchs M., Thacker L.R., Gilles H., White M.B., Unser A., Hovermale J., Gavis E., Noble N.A., Wade J.B. (2014). Validation of EncephalApp, smart- phone-based stroop test, for the diagnosis of covert hepatic encephalopathy. Clin. Gastroenterol. Hepatol..

[22] Bajaj J.S., Thacker L.R., Heuman D.M., Fuchs M., Sterling R.K., Sanyal A.J., Puri P., Siddiqui M.S., Stravitz R.T., Bouneva I., Luketic V., Noble N., White M.B., Monteith P., Unser A., Wade J.B. (2013). The Stroop smartphone application is a short and valid method to screen for minimal hepatic encephalopathy. Hepatology.

[23] Chowriappa A., Raza S.J., Fazili A., Field E., Malito C., Samarasekera D., Shi Y., Ahmed K., Wilding G., Kaouk J., Eun D.D., Ghazi A., Peabody J.O., Kesavadas T., Mohler J.L., Guru K.A. (2015). Augmented-reality-based skills training for robot-assisted urethrovesical anastomosis: a multi-institutional randomised controlled trial. BJU Int..

[24] R Core Team (2021). https://www.R-project.org/.

[25] Wickham H., Averick M., Bryan J., Chang W., D'Agostino McGowan L., Francois R., Francois G., Hayes A., Henry L., Hester J., Kuhn M., Pedersen T.L., Miller E., Bache S.M., Muller K., Ooms J., Robinson D., Seidel D.P., Spinu V., Takahashi K., Vaughan D., Wilke C., Woo K., Yutani H. (2019). Welcome to the tidyverse. J. Open Source Softw..

[26] Sjoberg D.D., Whiting K., Curry M., Lavery J.A., Larmarange J. (2021). Reproducible summary tables with the gtsummary package. The R Journal.

[27] Akiba T., Nakada T., Inagaki T. (2015). Simulation of the fissureless technique for thoracoscopic segmentectomy using rapid prototyping. Ann. Thorac. Cardiovasc. Surg..

[28] Zheng Y.X., Yu D.F., Zhao J.G., Wu Y.L., Zheng B. (2016). 3D printout models vs 3D-rendered images: which is better for preoperative planning?. J. Surg. Educ..

[29] Liu X., Zhao Y., Xuan Y., Lan X., Zhao J., Lan X., Han B., Jiao W. (2019 Dec). Three-dimensional printing in the preoperative planning of thoracoscopic pulmonary segmentectomy. Transl. Lung Cancer Res..

[30] Qiu B., Ji Y., He H., Zhao J., Xue Q., Gao S. (2020). Three-dimensional reconstruction/personalized three-dimensional printed model for thoracoscopic anatomical partial-lobectomy in stage I lung cancer: a retrospective study. Transl. Lung Cancer Res..

[31] O'Donnell R.D., Eggemeier F.T., Boff K.R., Kaufman L., Thomas J.P. (1986). Handbook of Perception and Performance.

[32] Carswell C.M., Clarke D., Seales W.B. (2005). Assessing mental workload during laparoscopic surgery. Surg Innov.

[33] Yurko Y.Y., Scerbo M.W., Prabhu A.S., Acker C.E., Stefanidis D. (2010). Higher mental workload is associated with poorer laparoscopic performance as measured by the NASA-TLX tool. Simul Healthc.

[34] Hoffman E., Pene N., Rognin L., Zeghal K. (2003). Introducing a new spacing instruction. Impact of spacing tolerance on flight crew activity. Proc. Hum. Factors Ergon. Soc. Annu. Meet..

[35] Grier R., Wickens C., Kaber D., Strayer D., Boehm-Davis D., Trafton G., St John M. (2008). The red-line of workload: theory, research, and design. Proc. Hum. Factors Ergon. Soc. Annu. Meet..

[36] Mazur L.M., Mosaly P.R., Hoyle L.M., Jones E.L., Chera B.S., Marks L.B. (2014). Relating physician's workload with errors during radiation therapy planning. Pract Radiat Oncol.

[37] Bates M.E., Lemay E.P. (2004). The d2 Test of attention: construct validity and extensions in scoring techniques. J. Int. Neuropsychol. Soc..

[38] Schmidt-Atzert L., Krumm S., Bühner M. (2008). Aufmerksamkeitsdiagnostik: Ableitung eines Struk- turmodells und systematische Einordnung von Tests. Z. für Neuropsychol..

